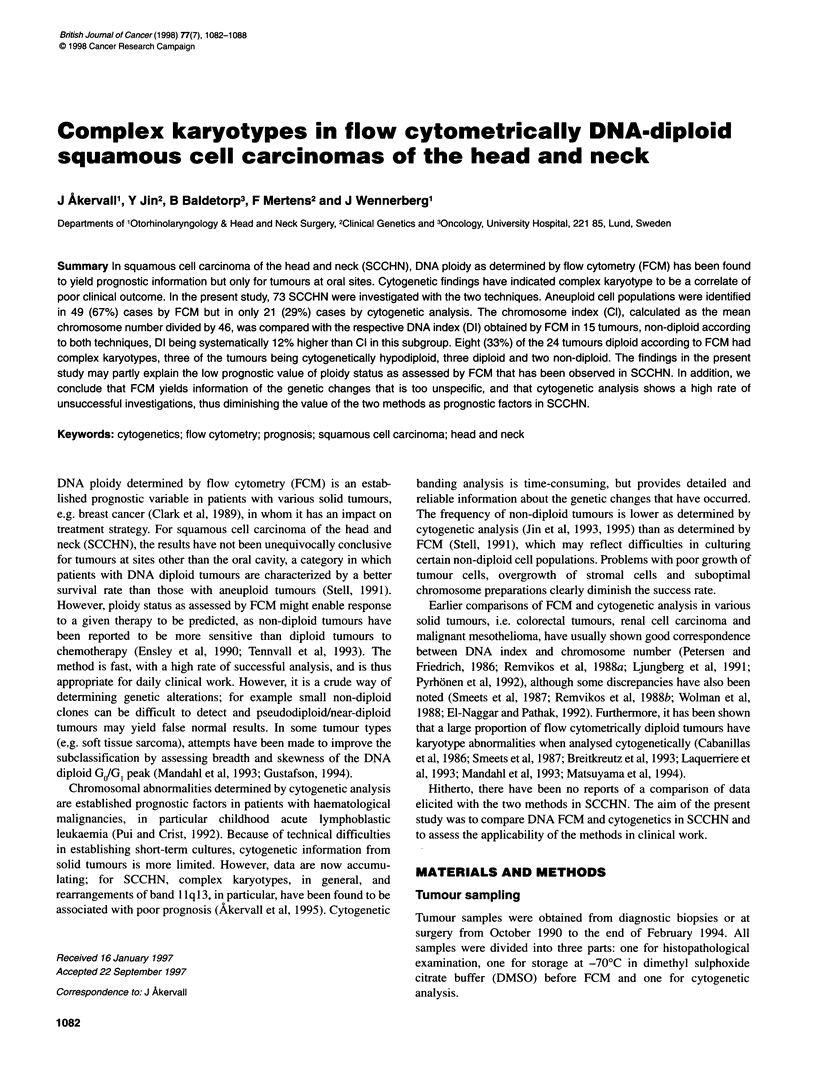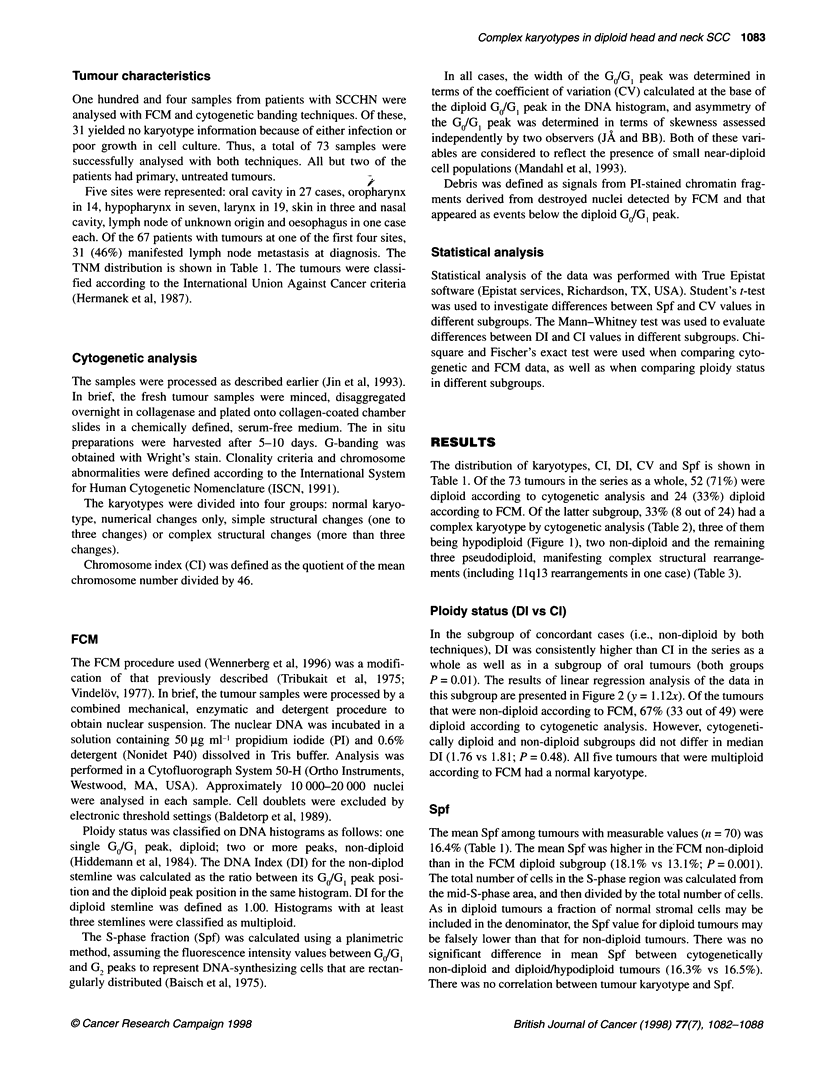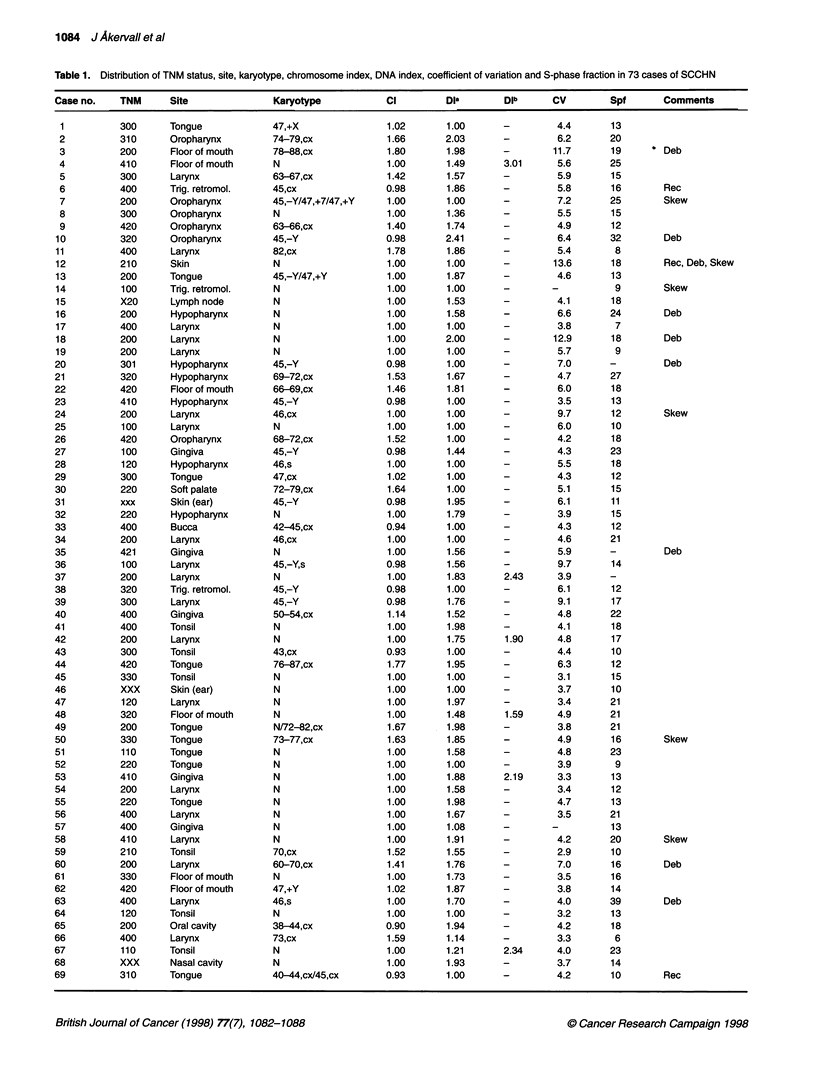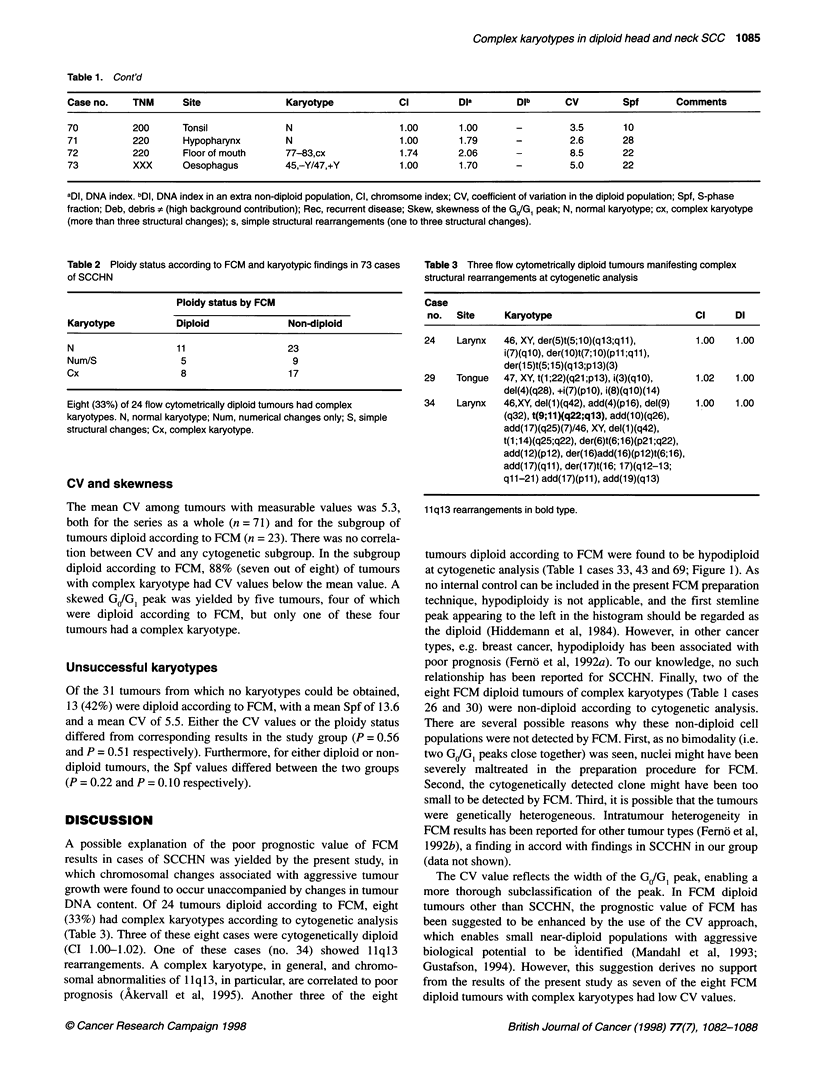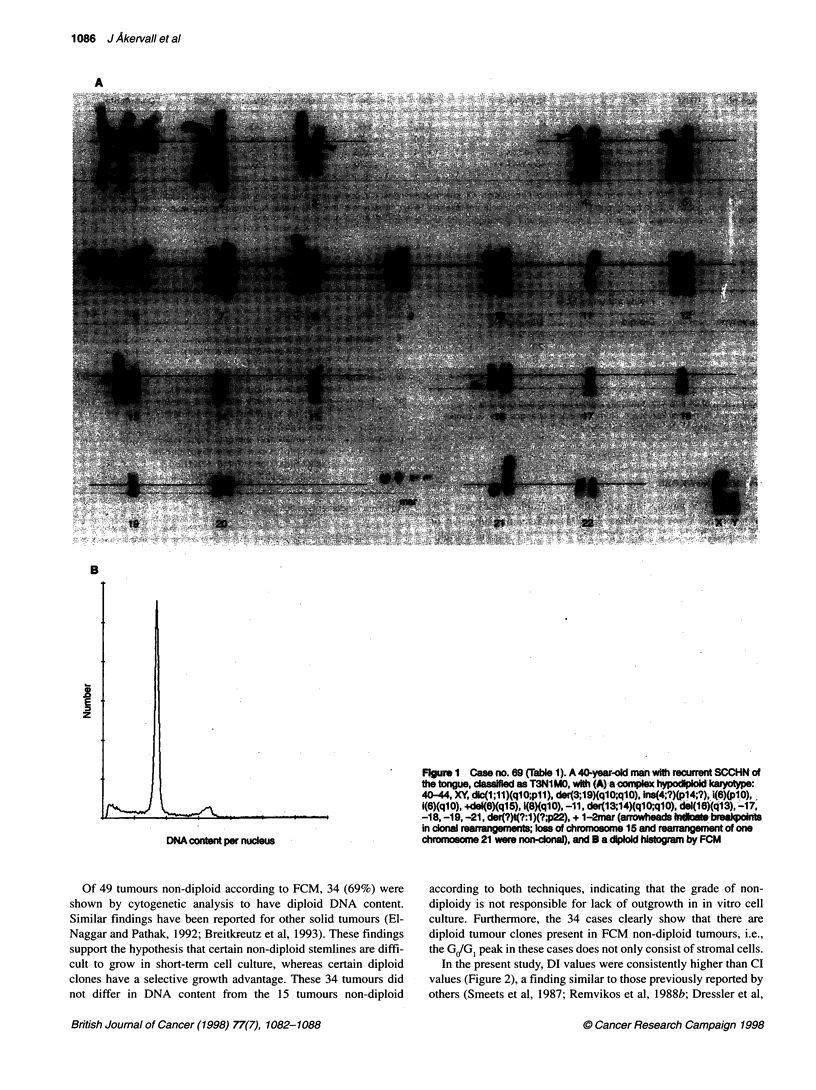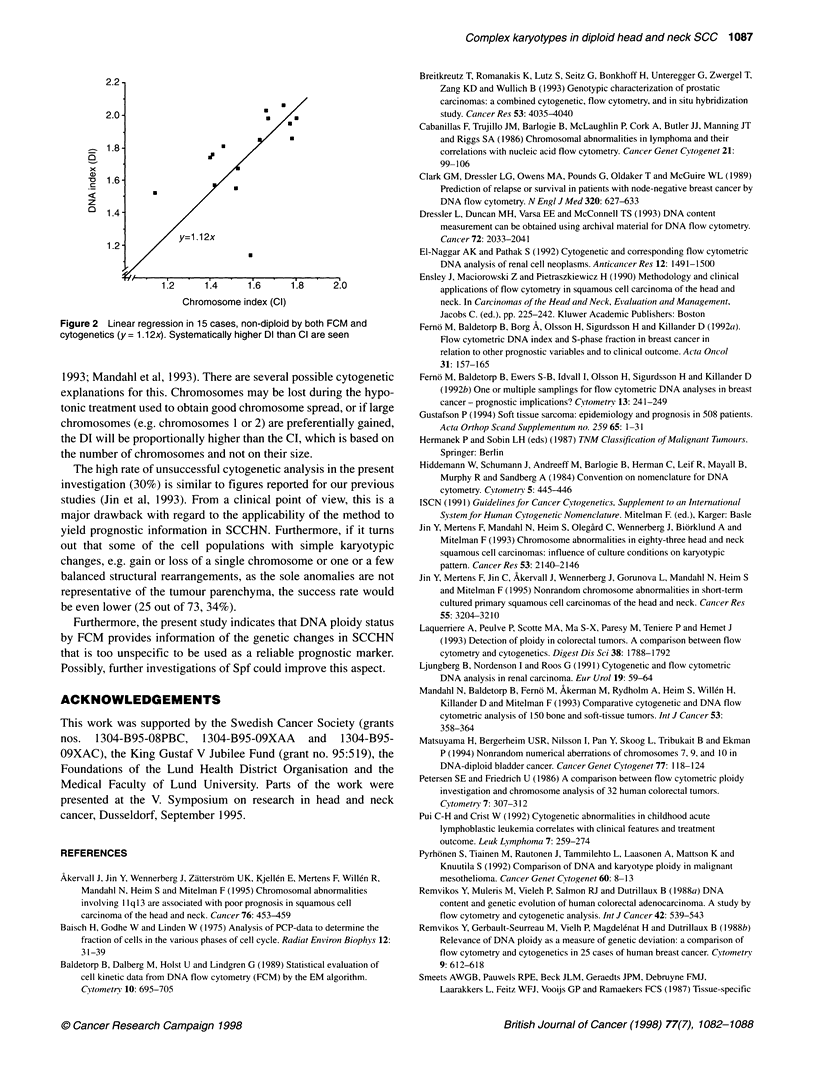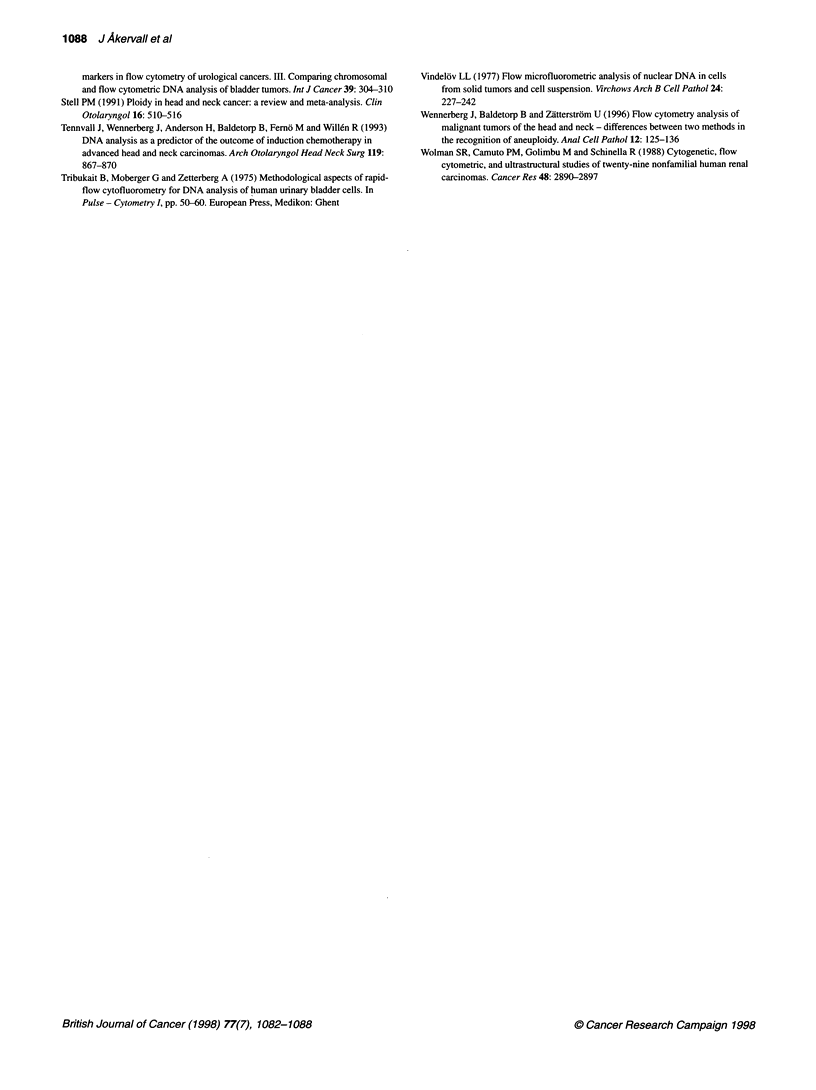# Complex karyotypes in flow cytometrically DNA-diploid squamous cell carcinomas of the head and neck.

**DOI:** 10.1038/bjc.1998.180

**Published:** 1998-04

**Authors:** J. Akervall, Y. Jin, B. Baldetorp, F. Mertens, J. Wennerberg

**Affiliations:** Department of Otorhinolaryngology & Head and Neck Surgery, University Hospital, Lund, Sweden.

## Abstract

**Images:**


British Journal of Cancer (1998) 77(7), 1082–1088
© 1998 Cancer Research Campaign

### Complex karyotypes in flow cytometrically DNA-diploid squamous cell carcinomas of the head and neck

**J Åkervall[1], Y Jin[2], B Baldetorp[3], F Mertens[2] and J Wennerberg[1]**

Departments of [1]Otorhinolaryngology & Head and Neck Surgery, [2]Clinical Genetics and [3]Oncology, University Hospital, 221 85, Lund, Sweden

**Summary** In squamous cell carcinoma of the head and neck (SCCHN), DNA ploidy as determined by flow cytometry (FCM) has been found to yield prognostic information but only for tumours at oral sites. Cytogenetic findings have indicated complex karyotype to be a correlate of poor clinical outcome. In the present study, 73 SCCHN were investigated with the two techniques. Aneuploid cell populations were identified in 49 (67%) cases by FCM but in only 21 (29%) cases by cytogenetic analysis. The chromosome index (CI), calculated as the mean chromosome number divided by 46, was compared with the respective DNA index (DI) obtained by FCM in 15 tumours, non-diploid according to both techniques, DI being systematically 12% higher than CI in this subgroup. Eight (33%) of the 24 tumours diploid according to FCM had complex karyotypes, three of the tumours being cytogenetically hypodiploid, three diploid and two non-diploid. The findings in the present study may partly explain the low prognostic value of ploidy status as assessed by FCM that has been observed in SCCHN. In addition, we conclude that FCM yields information of the genetic changes that is too unspecific, and that cytogenetic analysis shows a high rate of unsuccessful investigations, thus diminishing the value of the two methods as prognostic factors in SCCHN.

**Keywords:** cytogenetics; flow cytometry; prognosis; squamous cell carcinoma; head and neck

DNA ploidy determined by flow cytometry (FCM) is an established prognostic variable in patients with various solid tumours, e.g. breast cancer (Clark et al, 1989), in whom it has an impact on treatment strategy. For squamous cell carcinoma of the head and neck (SCCHN), the results have not been unequivocally conclusive for tumours at sites other than the oral cavity, a category in which patients with DNA diploid tumours are characterized by a better survival rate than those with aneuploid tumours (Stell, 1991). However, ploidy status as assessed by FCM might enable response to a given therapy to be predicted, as non-diploid tumours have been reported to be more sensitive than diploid tumours to chemotherapy (Ensley et al, 1990; Tennvall et al, 1993). The method is fast, with a high rate of successful analysis, and is thus appropriate for daily clinical work. However, it is a crude way of determining genetic alterations; for example small non-diploid clones can be difficult to detect and pseudodiploid/near-diploid tumours may yield false normal results. In some tumour types (e.g. soft tissue sarcoma), attempts have been made to improve the subclassification by assessing breadth and skewness of the DNA diploid $G_0/G_1$ peak (Mandahl et al, 1993; Gustafson, 1994).

Chromosomal abnormalities determined by cytogenetic analysis are established prognostic factors in patients with haematological malignancies, in particular childhood acute lymphoblastic leukaemia (Pui and Crist, 1992). Because of technical difficulties in establishing short-term cultures, cytogenetic information from solid tumours is more limited. However, data are now accumulating; for SCCHN, complex karyotypes, in general, and rearrangements of band 11q13, in particular, have been found to be associated with poor prognosis (Åkervall et al, 1995). Cytogenetic banding analysis is time-consuming, but provides detailed and reliable information about the genetic changes that have occurred. The frequency of non-diploid tumours is lower as determined by cytogenetic analysis (Jin et al, 1993, 1995) than as determined by FCM (Stell, 1991), which may reflect difficulties in culturing certain non-diploid cell populations. Problems with poor growth of tumour cells, overgrowth of stromal cells and suboptimal chromosome preparations clearly diminish the success rate.

Earlier comparisons of FCM and cytogenetic analysis in various solid tumours, i.e. colorectal tumours, renal cell carcinoma and malignant mesothelioma, have usually shown good correspondence between DNA index and chromosome number (Petersen and Friedrich, 1986; Remvikos et al, 1988*a*; Ljungberg et al, 1991; Pyrhönen et al, 1992), although some discrepancies have also been noted (Smeets et al, 1987; Remvikos et al, 1988*b*; Wolman et al, 1988; El-Naggar and Pathak, 1992). Furthermore, it has been shown that a large proportion of flow cytometrically diploid tumours have karyotype abnormalities when analysed cytogenetically (Cabanillas et al, 1986; Smeets et al, 1987; Breitkreutz et al, 1993; Laquerriere et al, 1993; Mandahl et al, 1993; Matsuyama et al, 1994).

Hitherto, there have been no reports of a comparison of data elicited with the two methods in SCCHN. The aim of the present study was to compare DNA FCM and cytogenetics in SCCHN and to assess the applicability of the methods in clinical work.

### MATERIALS AND METHODS

### Tumour sampling

Tumour samples were obtained from diagnostic biopsies or at surgery from October 1990 to the end of February 1994. All samples were divided into three parts: one for histopathological examination, one for storage at –70°C in dimethyl sulphoxide citrate buffer (DMSO) before FCM and one for cytogenetic analysis.

*Received 16 January 1997*
*Accepted 22 September 1997*

*Correspondence to:* J Åkervall

### Tumour characteristics

One hundred and four samples from patients with SCCHN were analysed with FCM and cytogenetic banding techniques. Of these, 31 yielded no karyotype information because of either infection or poor growth in cell culture. Thus, a total of 73 samples were successfully analysed with both techniques. All but two of the patients had primary, untreated tumours.

Five sites were represented: oral cavity in 27 cases, oropharynx in 14, hypopharynx in seven, larynx in 19, skin in three and nasal cavity, lymph node of unknown origin and oesophagus in one case each. Of the 67 patients with tumours at one of the first four sites, 31 (46%) manifested lymph node metastasis at diagnosis. The TNM distribution is shown in Table 1. The tumours were classified according to the International Union Against Cancer criteria (Hermanek et al, 1987).

### Cytogenetic analysis

The samples were processed as described earlier (Jin et al, 1993). In brief, the fresh tumour samples were minced, disaggregated overnight in collagenase and plated onto collagen-coated chamber slides in a chemically defined, serum-free medium. The in situ preparations were harvested after 5–10 days. G-banding was obtained with Wright's stain. Clonality criteria and chromosome abnormalities were defined according to the International System for Human Cytogenetic Nomenclature (ISCN, 1991).

The karyotypes were divided into four groups: normal karyotype, numerical changes only, simple structural changes (one to three changes) or complex structural changes (more than three changes).

Chromosome index (CI) was defined as the quotient of the mean chromosome number divided by 46.

### FCM

The FCM procedure used (Wennerberg et al, 1996) was a modification of that previously described (Tribukait et al, 1975; Vindelöv, 1977). In brief, the tumour samples were processed by a combined mechanical, enzymatic and detergent procedure to obtain nuclear suspension. The nuclear DNA was incubated in a solution containing 50 μg ml$^{-1}$ propidium iodide (PI) and 0.6% detergent (Nonidet P40) dissolved in Tris buffer. Analysis was performed in a Cytofluorograph System 50-H (Ortho Instruments, Westwood, MA, USA). Approximately 10 000–20 000 nuclei were analysed in each sample. Cell doublets were excluded by electronic threshold settings (Baldetorp et al, 1989).

Ploidy status was classified on DNA histograms as follows: one single $G_0/G_1$ peak, diploid; two or more peaks, non-diploid (Hiddemann et al, 1984). The DNA Index (DI) for the non-diplod stemline was calculated as the ratio between its $G_0/G_1$ peak position and the diploid peak position in the same histogram. DI for the diploid stemline was defined as 1.00. Histograms with at least three stemlines were classified as multiploid.

The S-phase fraction (Spf) was calculated using a planimetric method, assuming the fluorescence intensity values between $G_0/G_1$ and $G_2$ peaks to represent DNA-synthesizing cells that are rectangularly distributed (Baisch et al, 1975).

In all cases, the width of the $G_0/G_1$ peak was determined in terms of the coefficient of variation (CV) calculated at the base of the diploid $G_0/G_1$ peak in the DNA histogram, and asymmetry of the $G_0/G_1$ peak was determined in terms of skewness assessed independently by two observers (JÅ and BB). Both of these variables are considered to reflect the presence of small near-diploid cell populations (Mandahl et al, 1993).

Debris was defined as signals from PI-stained chromatin fragments derived from destroyed nuclei detected by FCM and that appeared as events below the diploid $G_0/G_1$ peak.

### Statistical analysis

Statistical analysis of the data was performed with True Epistat software (Epistat services, Richardson, TX, USA). Student's *t*-test was used to investigate differences between Spf and CV values in different subgroups. The Mann–Whitney test was used to evaluate differences between DI and CI values in different subgroups. Chi-square and Fischer's exact test were used when comparing cytogenetic and FCM data, as well as when comparing ploidy status in different subgroups.

### RESULTS

The distribution of karyotypes, CI, DI, CV and Spf is shown in Table 1. Of the 73 tumours in the series as a whole, 52 (71%) were diploid according to cytogenetic analysis and 24 (33%) diploid according to FCM. Of the latter subgroup, 33% (8 out of 24) had a complex karyotype by cytogenetic analysis (Table 2), three of them being hypodiploid (Figure 1), two non-diploid and the remaining three pseudodiploid, manifesting complex structural rearrangements (including 11q13 rearrangements in one case) (Table 3).

### Ploidy status (DI vs CI)

In the subgroup of concordant cases (i.e., non-diploid by both techniques), DI was consistently higher than CI in the series as a whole as well as in a subgroup of oral tumours (both groups $P = 0.01$). The results of linear regression analysis of the data in this subgroup are presented in Figure 2 ($y = 1.12x$). Of the tumours that were non-diploid according to FCM, 67% (33 out of 49) were diploid according to cytogenetic analysis. However, cytogenetically diploid and non-diploid subgroups did not differ in median DI (1.76 vs 1.81; $P = 0.48$). All five tumours that were multiploid according to FCM had a normal karyotype.

### Spf

The mean Spf among tumours with measurable values ($n = 70$) was 16.4% (Table 1). The mean Spf was higher in the FCM non-diploid than in the FCM diploid subgroup (18.1% vs 13.1%; $P = 0.001$). The total number of cells in the S-phase region was calculated from the mid-S-phase area, and then divided by the total number of cells. As in diploid tumours a fraction of normal stromal cells may be included in the denominator, the Spf value for diploid tumours may be falsely lower than that for non-diploid tumours. There was no significant difference in mean Spf between cytogenetically non-diploid and diploid/hypodiploid tumours (16.3% vs 16.5%). There was no correlation between tumour karyotype and Spf.

© Cancer Research Campaign 1998

**Table 1.** Distribution of TNM status, site, karyotype, chromosome index, DNA index, coefficient of variation and S-phase fraction in 73 cases of SCCHN

| Case no. | TNM | Site | Karyotype | CI | DI[a] | DI[b] | CV | Spf | Comments |
|---|---|---|---|---|---|---|---|---|---|
| 1 | 300 | Tongue | 47,+X | 1.02 | 1.00 | – | 4.4 | 13 | |
| 2 | 310 | Oropharynx | 74–79,cx | 1.66 | 2.03 | – | 6.2 | 20 | |
| 3 | 200 | Floor of mouth | 78–88,cx | 1.80 | 1.98 | – | 11.7 | 19 | • Deb |
| 4 | 410 | Floor of mouth | N | 1.00 | 1.49 | 3.01 | 5.6 | 25 | |
| 5 | 300 | Larynx | 63–67,cx | 1.42 | 1.57 | – | 5.9 | 15 | |
| 6 | 400 | Trig. retromol. | 45,cx | 0.98 | 1.86 | – | 5.8 | 16 | Rec |
| 7 | 200 | Oropharynx | 45,–Y/47,+7/47,+Y | 1.00 | 1.00 | – | 7.2 | 25 | Skew |
| 8 | 300 | Oropharynx | N | 1.00 | 1.36 | – | 5.5 | 15 | |
| 9 | 420 | Oropharynx | 63–66,cx | 1.40 | 1.74 | – | 4.9 | 12 | |
| 10 | 320 | Oropharynx | 45,–Y | 0.98 | 2.41 | – | 6.4 | 32 | Deb |
| 11 | 400 | Larynx | 82,cx | 1.78 | 1.86 | – | 5.4 | 8 | |
| 12 | 210 | Skin | N | 1.00 | 1.00 | – | 13.6 | 18 | Rec, Deb, Skew |
| 13 | 200 | Tongue | 45,–Y/47,+Y | 1.00 | 1.87 | – | 4.6 | 13 | |
| 14 | 100 | Trig. retromol. | N | 1.00 | 1.00 | – | – | 9 | Skew |
| 15 | X20 | Lymph node | N | 1.00 | 1.53 | – | 4.1 | 18 | |
| 16 | 200 | Hypopharynx | N | 1.00 | 1.58 | – | 6.6 | 24 | Deb |
| 17 | 400 | Larynx | N | 1.00 | 1.00 | – | 3.8 | 7 | |
| 18 | 200 | Larynx | N | 1.00 | 2.00 | – | 12.9 | 18 | Deb |
| 19 | 200 | Larynx | N | 1.00 | 1.00 | – | 5.7 | 9 | |
| 20 | 301 | Hypopharynx | 45,–Y | 0.98 | 1.00 | – | 7.0 | – | Deb |
| 21 | 320 | Hypopharynx | 69–72,cx | 1.53 | 1.67 | – | 4.7 | 27 | |
| 22 | 420 | Floor of mouth | 66–69,cx | 1.46 | 1.81 | – | 6.0 | 18 | |
| 23 | 410 | Hypopharynx | 45,–Y | 0.98 | 1.00 | – | 3.5 | 13 | |
| 24 | 200 | Larynx | 46,cx | 1.00 | 1.00 | – | 9.7 | 12 | Skew |
| 25 | 100 | Larynx | N | 1.00 | 1.00 | – | 6.0 | 10 | |
| 26 | 420 | Oropharynx | 68–72,cx | 1.52 | 1.00 | – | 4.2 | 18 | |
| 27 | 100 | Gingiva | 45,–Y | 0.98 | 1.44 | – | 4.3 | 23 | |
| 28 | 120 | Hypopharynx | 46,s | 1.00 | 1.00 | – | 5.5 | 18 | |
| 29 | 300 | Tongue | 47,cx | 1.02 | 1.00 | – | 4.3 | 12 | |
| 30 | 220 | Soft palate | 72–79,cx | 1.64 | 1.00 | – | 5.1 | 15 | |
| 31 | xxx | Skin (ear) | 45,–Y | 0.98 | 1.95 | – | 6.1 | 11 | |
| 32 | 220 | Hypopharynx | N | 1.00 | 1.79 | – | 3.9 | 15 | |
| 33 | 400 | Bucca | 42–45,cx | 0.94 | 1.00 | – | 4.3 | 12 | |
| 34 | 200 | Larynx | 46,cx | 1.00 | 1.00 | – | 4.6 | 21 | |
| 35 | 421 | Gingiva | N | 1.00 | 1.56 | – | 5.9 | – | Deb |
| 36 | 100 | Larynx | 45,–Y,s | 0.98 | 1.56 | – | 9.7 | 14 | |
| 37 | 200 | Larynx | N | 1.00 | 1.83 | 2.43 | 3.9 | – | |
| 38 | 320 | Trig. retromol. | 45,–Y | 0.98 | 1.00 | – | 6.1 | 12 | |
| 39 | 300 | Larynx | 45,–Y | 0.98 | 1.76 | – | 9.1 | 17 | |
| 40 | 400 | Gingiva | 50–54,cx | 1.14 | 1.52 | – | 4.8 | 22 | |
| 41 | 400 | Tonsil | N | 1.00 | 1.98 | – | 4.1 | 18 | |
| 42 | 200 | Larynx | N | 1.00 | 1.75 | 1.90 | 4.8 | 17 | |
| 43 | 300 | Tonsil | 43,cx | 0.93 | 1.00 | – | 4.4 | 10 | |
| 44 | 420 | Tongue | 76–87,cx | 1.77 | 1.95 | – | 6.3 | 12 | |
| 45 | 330 | Tonsil | N | 1.00 | 1.00 | – | 3.1 | 15 | |
| 46 | XXX | Skin (ear) | N | 1.00 | 1.00 | – | 3.7 | 10 | |
| 47 | 120 | Larynx | N | 1.00 | 1.97 | – | 3.4 | 21 | |
| 48 | 320 | Floor of mouth | N | 1.00 | 1.48 | 1.59 | 4.9 | 21 | |
| 49 | 200 | Tongue | N/72–82,cx | 1.67 | 1.98 | – | 3.8 | 21 | |
| 50 | 330 | Tongue | 73–77,cx | 1.63 | 1.85 | – | 4.9 | 16 | Skew |
| 51 | 110 | Tongue | N | 1.00 | 1.58 | – | 4.8 | 23 | |
| 52 | 220 | Tongue | N | 1.00 | 1.00 | – | 3.9 | 9 | |
| 53 | 410 | Gingiva | N | 1.00 | 1.88 | 2.19 | 3.3 | 13 | |
| 54 | 200 | Larynx | N | 1.00 | 1.58 | – | 3.4 | 12 | |
| 55 | 220 | Tongue | N | 1.00 | 1.98 | – | 4.7 | 13 | |
| 56 | 400 | Larynx | N | 1.00 | 1.67 | – | 3.5 | 21 | |
| 57 | 400 | Gingiva | N | 1.00 | 1.08 | – | – | 13 | |
| 58 | 410 | Larynx | N | 1.00 | 1.91 | – | 4.2 | 20 | Skew |
| 59 | 210 | Tonsil | 70,cx | 1.52 | 1.55 | – | 2.9 | 10 | |
| 60 | 200 | Larynx | 60–70,cx | 1.41 | 1.76 | – | 7.0 | 16 | Deb |
| 61 | 330 | Floor of mouth | N | 1.00 | 1.73 | – | 3.5 | 16 | |
| 62 | 420 | Floor of mouth | 47,+Y | 1.02 | 1.87 | – | 3.8 | 14 | |
| 63 | 400 | Larynx | 46,s | 1.00 | 1.70 | – | 4.0 | 39 | Deb |
| 64 | 120 | Tonsil | N | 1.00 | 1.00 | – | 3.2 | 13 | |
| 65 | 200 | Oral cavity | 38–44,cx | 0.90 | 1.94 | – | 4.2 | 18 | |
| 66 | 400 | Larynx | 73,cx | 1.59 | 1.14 | – | 3.3 | 6 | |
| 67 | 110 | Tonsil | N | 1.00 | 1.21 | 2.34 | 4.0 | 23 | |
| 68 | XXX | Nasal cavity | N | 1.00 | 1.93 | – | 3.7 | 14 | |
| 69 | 310 | Tongue | 40–44,cx/45,cx | 0.93 | 1.00 | – | 4.2 | 10 | Rec |

© Cancer Research Campaign 1998

**Table 1.** *Cont'd*

| Case no. | TNM | Site | Karyotype | CI | DI[a] | DI[b] | CV | Spf | Comments |
|---|---|---|---|---|---|---|---|---|---|
| 70 | 200 | Tonsil | N | 1.00 | 1.00 | – | 3.5 | 10 | |
| 71 | 220 | Hypopharynx | N | 1.00 | 1.79 | – | 2.6 | 28 | |
| 72 | 220 | Floor of mouth | 77–83,cx | 1.74 | 2.06 | – | 8.5 | 22 | |
| 73 | XXX | Oesophagus | 45,–Y/47,+Y | 1.00 | 1.70 | – | 5.0 | 22 | |

[a]DI, DNA index. [b]DI, DNA index in an extra non-diploid population, CI, chromsome index; CV, coefficient of variation in the diploid population; Spf, S-phase fraction; Deb, debris ≠ (high background contribution); Rec, recurrent disease; Skew, skewness of the $G_0/G_1$ peak; N, normal karyotype; cx, complex karyotype (more than three structural changes); s, simple structural rearrangements (one to three structural changes).

**Table 2** Ploidy status according to FCM and karyotypic findings in 73 cases of SCCHN

| | Ploidy status by FCM | |
|---|---|---|
| Karyotype | Diploid | Non-diploid |
| N | 11 | 23 |
| Num/S | 5 | 9 |
| Cx | 8 | 17 |

Eight (33%) of 24 flow cytometrically diploid tumours had complex karyotypes. N, normal karyotype; Num, numerical changes only; S, simple structural changes; Cx, complex karyotype.

### CV and skewness

The mean CV among tumours with measurable values was 5.3, both for the series as a whole ($n = 71$) and for the subgroup of tumours diploid according to FCM ($n = 23$). There was no correlation between CV and any cytogenetic subgroup. In the subgroup diploid according to FCM, 88% (seven out of eight) of tumours with complex karyotype had CV values below the mean value. A skewed $G_0/G_1$ peak was yielded by five tumours, four of which were diploid according to FCM, but only one of these four tumours had a complex karyotype.

### Unsuccessful karyotypes

Of the 31 tumours from which no karyotypes could be obtained, 13 (42%) were diploid according to FCM, with a mean Spf of 13.6 and a mean CV of 5.5. Either the CV values or the ploidy status differed from corresponding results in the study group ($P = 0.56$ and $P = 0.51$ respectively). Furthermore, for either diploid or non-diploid tumours, the Spf values differed between the two groups ($P = 0.22$ and $P = 0.10$ respectively).

### DISCUSSION

A possible explanation of the poor prognostic value of FCM results in cases of SCCHN was yielded by the present study, in which chromosomal changes associated with aggressive tumour growth were found to occur unaccompanied by changes in tumour DNA content. Of 24 tumours diploid according to FCM, eight (33%) had complex karyotypes according to cytogenetic analysis (Table 3). Three of these eight cases were cytogenetically diploid (CI 1.00–1.02). One of these cases (no. 34) showed 11q13 rearrangements. A complex karyotype, in general, and chromosomal abnormalities of 11q13, in particular, are correlated to poor prognosis (Åkervall et al, 1995). Another three of the eight

**Table 3** Three flow cytometrically diploid tumours manifesting complex structural rearrangements at cytogenetic analysis

| Case no. | Site | Karyotype | CI | DI |
|---|---|---|---|---|
| 24 | Larynx | 46, XY, der(5)t(5;10)(q13;q11), i(7)(q10), der(10)t(7;10)(p11;q11), der(15)t(5;15)(q13;p13)(3) | 1.00 | 1.00 |
| 29 | Tongue | 47, XY, t(1;22)(q21;p13), i(3)(q10), del(4)(q28), +i(7)(p10), i(8)(q10)(14) | 1.02 | 1.00 |
| 34 | Larynx | 46,XY, del(1)(q42), add(4)(p16), del(9)(q32), **t(9;11)(q22;q13)**, add(10)(q26), add(17)(q25)(7)/46, XY, del(1)(q42), t(1;14)(q25;q22), der(6)t(6;16)(p21;q22), add(12)(p12), der(16)add(16)(p12)t(6;16), add(17)(q11), der(17)t(16; 17)(q12–13; q11–21) add(17)(p11), add(19)(q13) | 1.00 | 1.00 |

11q13 rearrangements in bold type.

tumours diploid according to FCM were found to be hypodiploid at cytogenetic analysis (Table 1 cases 33, 43 and 69; Figure 1). As no internal control can be included in the present FCM preparation technique, hypodiploidy is not applicable, and the first stemline peak appearing to the left in the histogram should be regarded as the diploid (Hiddemann et al, 1984). However, in other cancer types, e.g. breast cancer, hypodiploidy has been associated with poor prognosis (Fernö et al, 1992*a*). To our knowledge, no such relationship has been reported for SCCHN. Finally, two of the eight FCM diploid tumours of complex karyotypes (Table 1 cases 26 and 30) were non-diploid according to cytogenetic analysis. There are several possible reasons why these non-diploid cell populations were not detected by FCM. First, as no bimodality (i.e. two $G_0/G_1$ peaks close together) was seen, nuclei might have been severely maltreated in the preparation procedure for FCM. Second, the cytogenetically detected clone might have been too small to be detected by FCM. Third, it is possible that the tumours were genetically heterogeneous. Intratumour heterogeneity in FCM results has been reported for other tumour types (Fernö et al, 1992*b*), a finding in accord with findings in SCCHN in our group (data not shown).

The CV value reflects the width of the $G_0/G_1$ peak, enabling a more thorough subclassification of the peak. In FCM diploid tumours other than SCCHN, the prognostic value of FCM has been suggested to be enhanced by the use of the CV approach, which enables small near-diploid populations with aggressive biological potential to be identified (Mandahl et al, 1993; Gustafson, 1994). However, this suggestion derives no support from the results of the present study as seven of the eight FCM diploid tumours with complex karyotypes had low CV values.

© Cancer Research Campaign 1998

**Figure 1** Case no. 69 (Table 1). A 40-year-old man with recurrent SCCHN of the tongue, classified as T3N1M0, with (**A**) a complex hypodiploid karyotype: 40–44, XY, dic(1;11)(q10;p11), der(3;19)(q10;q10), ins(4;?)(p14;?), i(6)(p10), i(6)(q10), +del(6)(q15), i(8)(q10), −11, der(13;14)(q10;q10), del(16)(q13), −17, −18, −19, −21, der(?)t(?:1)(?;p22), + 1–2mar (arrowheads indicate breakpoints in clonal rearrangements; loss of chromosome 15 and rearrangement of one chromosome 21 were non-clonal), and **B** a diploid histogram by FCM

Of 49 tumours non-diploid according to FCM, 34 (69%) were shown by cytogenetic analysis to have diploid DNA content. Similar findings have been reported for other solid tumours (El-Naggar and Pathak, 1992; Breitkreutz et al, 1993). These findings support the hypothesis that certain non-diploid stemlines are difficult to grow in short-term cell culture, whereas certain diploid clones have a selective growth advantage. These 34 tumours did not differ in DNA content from the 15 tumours non-diploid according to both techniques, indicating that the grade of non-diploidy is not responsible for lack of outgrowth in in vitro cell culture. Furthermore, the 34 cases clearly show that there are diploid tumour clones present in FCM non-diploid tumours, i.e., the $G_0/G_1$ peak in these cases does not only consist of stromal cells.

In the present study, DI values were consistently higher than CI values (Figure 2), a finding similar to those previously reported by others (Smeets et al, 1987; Remvikos et al, 1988*b*; Dressler et al,

 © Cancer Research Campaign 1998

**Figure 2** Linear regression in 15 cases, non-diploid by both FCM and cytogenetics ($y = 1.12x$). Systematically higher DI than CI are seen

1993; Mandahl et al, 1993). There are several possible cytogenetic explanations for this. Chromosomes may be lost during the hypotonic treatment used to obtain good chromosome spread, or if large chromosomes (e.g. chromosomes 1 or 2) are preferentially gained, the DI will be proportionally higher than the CI, which is based on the number of chromosomes and not on their size.

The high rate of unsuccessful cytogenetic analysis in the present investigation (30%) is similar to figures reported for our previous studies (Jin et al, 1993). From a clinical point of view, this is a major drawback with regard to the applicability of the method to yield prognostic information in SCCHN. Furthermore, if it turns out that some of the cell populations with simple karyotypic changes, e.g. gain or loss of a single chromosome or one or a few balanced structural rearrangements, as the sole anomalies are not representative of the tumour parenchyma, the success rate would be even lower (25 out of 73, 34%).

Furthermore, the present study indicates that DNA ploidy status by FCM provides information of the genetic changes in SCCHN that is too unspecific to be used as a reliable prognostic marker. Possibly, further investigations of Spf could improve this aspect.

### ACKNOWLEDGEMENTS

This work was supported by the Swedish Cancer Society (grants nos. 1304-B95-08PBC, 1304-B95-09XAA and 1304-B95-09XAC), the King Gustaf V Jubilee Fund (grant no. 95:519), the Foundations of the Lund Health District Organisation and the Medical Faculty of Lund University. Parts of the work were presented at the V. Symposium on research in head and neck cancer, Dusseldorf, September 1995.

### REFERENCES

Åkervall J, Jin Y, Wennerberg J, Zätterström UK, Kjellén E, Mertens F, Willén R, Mandahl N, Heim S and Mitelman F (1995) Chromosomal abnormalities involving 11q13 are associated with poor prognosis in squamous cell carcinoma of the head and neck. *Cancer* **76**: 453–459

Baisch H, Godhe W and Linden W (1975) Analysis of PCP-data to determine the fraction of cells in the various phases of cell cycle. *Radiat Environ Biophys* **12**: 31–39

Baldetorp B, Dalberg M, Holst U and Lindgren G (1989) Statistical evaluation of cell kinetic data from DNA flow cytometry (FCM) by the EM algorithm. *Cytometry* **10**: 695–705

Breitkreutz T, Romanakis K, Lutz S, Seitz G, Bonkhoff H, Unteregger G, Zwergel T, Zang KD and Wullich B (1993) Genotypic characterization of prostatic carcinomas: a combined cytogenetic, flow cytometry, and in situ hybridization study. *Cancer Res* **53**: 4035–4040

Cabanillas F, Trujillo JM, Barlogie B, McLaughlin P, Cork A, Butler JJ, Manning JT and Riggs SA (1986) Chromosomal abnormalities in lymphoma and their correlations with nucleic acid flow cytometry. *Cancer Genet Cytogenet* **21**: 99–106

Clark GM, Dressler LG, Owens MA, Pounds G, Oldaker T and McGuire WL (1989) Prediction of relapse or survival in patients with node-negative breast cancer by DNA flow cytometry. *N Engl J Med* **320**: 627–633

Dressler L, Duncan MH, Varsa EE and McConnell TS (1993) DNA content measurement can be obtained using archival material for DNA flow cytometry. *Cancer* **72**: 2033–2041

El-Naggar AK and Pathak S (1992) Cytogenetic and corresponding flow cytometric DNA analysis of renal cell neoplasms. *Anticancer Res* **12**: 1491–1500

Ensley J, Maciorowski Z and Pietraszkiewicz H (1990) Methodology and clinical applications of flow cytometry in squamous cell carcinoma of the head and neck. In *Carcinomas of the Head and Neck, Evaluation and Management*, Jacobs C. (ed.), pp. 225–242. Kluwer Academic Publishers: Boston

Fernö M, Baldetorp B, Borg Å, Olsson H, Sigurdsson H and Killander D (1992*a*). Flow cytometric DNA index and S-phase fraction in breast cancer in relation to other prognostic variables and to clinical outcome. *Acta Oncol* **31**: 157–165

Fernö M, Baldetorp B, Ewers S-B, Idvall I, Olsson H, Sigurdsson H and Killander D (1992*b*) One or multiple samplings for flow cytometric DNA analyses in breast cancer – prognostic implications? *Cytometry* **13**: 241–249

Gustafson P (1994) Soft tissue sarcoma: epidemiology and prognosis in 508 patients. *Acta Orthop Scand Supplementum no. 259* **65**: 1–31

Hermanek P and Sobin LH (eds) (1987) *TNM Classification of Malignant Tumours*. Springer: Berlin

Hiddemann W, Schumann J, Andreeff M, Barlogie B, Herman C, Leif R, Mayall B, Murphy R and Sandberg A (1984) Convention on nomenclature for DNA cytometry. *Cytometry* **5**: 445–446

ISCN (1991) *Guidelines for Cancer Cytogenetics, Supplement to an International System for Human Cytogenetic Nomenclature*. Mitelman F. (ed.), Karger: Basle

Jin Y, Mertens F, Mandahl N, Heim S, Olegård C, Wennerberg J, Biörklund A and Mitelman F (1993) Chromosome abnormalities in eighty-three head and neck squamous cell carcinomas: influence of culture conditions on karyotypic pattern. *Cancer Res* **53**: 2140–2146

Jin Y, Mertens F, Jin C, Åkervall J, Wennerberg J, Gorunova L, Mandahl N, Heim S and Mitelman F (1995) Nonrandom chromosome abnormalities in short-term cultured primary squamous cell carcinomas of the head and neck. *Cancer Res* **55**: 3204–3210

Laquerriere A, Peulve P, Scotte MA, Ma S-X, Paresy M, Teniere P and Hemet J (1993) Detection of ploidy in colorectal tumors. A comparison between flow cytometry and cytogenetics. *Digest Dis Sci* **38**: 1788–1792

Ljungberg B, Nordenson I and Roos G (1991) Cytogenetic and flow cytometric DNA analysis in renal carcinoma. *Eur Urol* **19**: 59–64

Mandahl N, Baldetorp B, Fernö M, Åkerman M, Rydholm A, Heim S, Willén H, Killander D and Mitelman F (1993) Comparative cytogenetic and DNA flow cytometric analysis of 150 bone and soft-tissue tumors. *Int J Cancer* **53**: 358–364

Matsuyama H, Bergerheim USR, Nilsson I, Pan Y, Skoog L, Tribukait B and Ekman P (1994) Nonrandom numerical aberrations of chromosomes 7, 9, and 10 in DNA-diploid bladder cancer. *Cancer Genet Cytogenet* **77**: 118–124

Petersen SE and Friedrich U (1986) A comparison between flow cytometric ploidy investigation and chromosome analysis of 32 human colorectal tumors. *Cytometry* **7**: 307–312

Pui C-H and Crist W (1992) Cytogenetic abnormalities in childhood acute lymphoblastic leukemia correlates with clinical features and treatment outcome. *Leuk Lymphoma* **7**: 259–274

Pyrhönen S, Tiainen M, Rautonen J, Tammilehto L, Laasonen A, Mattson K and Knuutila S (1992) Comparison of DNA and karyotype ploidy in malignant mesothelioma. *Cancer Genet Cytogenet* **60**: 8–13

Remvikos Y, Muleris M, Vieleh P, Salmon RJ and Dutrillaux B (1988*a*) DNA content and genetic evolution of human colorectal adenocarcinoma. A study by flow cytometry and cytogenetic analysis. *Int J Cancer* **42**: 539–543

Remvikos Y, Gerbault-Seurreau M, Vielh P, Magdelénat H and Dutrillaux B (1988*b*) Relevance of DNA ploidy as a measure of genetic deviation: a comparison of flow cytometry and cytogenetics in 25 cases of human breast cancer. *Cytometry* **9**: 612–618

Smeets AWGB, Pauwels RPE, Beck JLM, Geraedts JPM, Debruyne FMJ, Laarakkers L, Feitz WFJ, Vooijs GP and Ramaekers FCS (1987) Tissue-specific

© Cancer Research Campaign 1998

markers in flow cytometry of urological cancers. III. Comparing chromosomal and flow cytometric DNA analysis of bladder tumors. *Int J Cancer* **39**: 304–310

Stell PM (1991) Ploidy in head and neck cancer: a review and meta-analysis. *Clin Otolaryngol* **16**: 510–516

Tennvall J, Wennerberg J, Anderson H, Baldetorp B, Fernö M and Willén R (1993) DNA analysis as a predictor of the outcome of induction chemotherapy in advanced head and neck carcinomas. *Arch Otolaryngol Head Neck Surg* **119**: 867–870

Tribukait B, Moberger G and Zetterberg A (1975) Methodological aspects of rapid-flow cytofluorometry for DNA analysis of human urinary bladder cells. In *Pulse – Cytometry I*, pp. 50–60. European Press, Medikon: Ghent

Vindelöv LL (1977) Flow microfluorometric analysis of nuclear DNA in cells from solid tumors and cell suspension. *Virchows Arch B Cell Pathol* **24**: 227–242

Wennerberg J, Baldetorp B and Zätterström U (1996) Flow cytometry analysis of malignant tumors of the head and neck – differences between two methods in the recognition of aneuploidy. *Anal Cell Pathol* **12**: 125–136

Wolman SR, Camuto PM, Golimbu M and Schinella R (1988) Cytogenetic, flow cytometric, and ultrastructural studies of twenty-nine nonfamilial human renal carcinomas. *Cancer Res* **48**: 2890–2897

© Cancer Research Campaign 1998